# Cyclodextrin-Based Metal-Organic Frameworks (CD-MOFs) in Pharmaceutics and Biomedicine

**DOI:** 10.3390/pharmaceutics10040271

**Published:** 2018-12-12

**Authors:** Yaoyao Han, Weicong Liu, Jianjing Huang, Shuowen Qiu, Huarui Zhong, Dong Liu, Jianqiang Liu

**Affiliations:** 1 Dongguan Key Laboratory of Drug Design and Formulation Technology, Key Laboratory of Research and Development of New Medical Materials of Guangdong Medical University, School of Pharmacy, Guangdong Medical University, Dongguan 523808, China; 1555508227@163.com (Y.H.); gdmulwc@126.com (W.L.); pharmacy_crystal@163.com (J.H.); QSW854768606@163.com (S.Q.); Huarui_Zhong@163.com (H.Z.); 2 Shenzhen Huachuang Bio-pharmaceutical Technology Co. Ltd., Shenzhen 518112, China

**Keywords:** CD-MOFs, inclusion technology, drug delivery, applications

## Abstract

Metal-organic frameworks (MOFs) show promising application in biomedicine and pharmaceutics owing to their extraordinarily high surface area, tunable pore size, and adjustable internal surface properties. However, MOFs are prepared from non-renewable or toxic materials, which limit their real-world applications. Cyclodextrins (CDs) are a typical natural and biodegradable cyclic oligosaccharide and are primarily used to enhance the aqueous solubility, safety, and bioavailability of drugs by virtue of its low toxicity and highly flexible structure, offering a peculiar ability to form CD/drug inclusions. A sophisticated strategy where CD is deployed as a ligand to form an assembly of cyclodextrin-based MOFs (CD-MOFs) may overcome real-world application drawbacks of MOFs. CD-MOFs incorporate the porous features of MOFs and the encapsulation capability of CD for drug molecules, leading to outstanding properties when compared with traditional hybrid materials. This review focuses on the inclusion technology and drug delivery properties associated with CD-MOFs. In addition, synthetic strategies and currently developed uses of CD-MOFs are highlighted as well. Also, perspectives and future challenges in this rapidly developing research area are discussed.

## 1. Introduction

The ongoing efforts for the commercialization of new bioactive molecules with low water solubility, poor stability, and/or comparatively short biological half-life are extremely slow, which is a challenge for researchers working in the field of pharmaceutics. Consequently, extensive research activities have concentrated on the development of carrier materials and delivery theories [1,2,3,4,5,6] that incorporate a controllable release process, high loading efficiency, low toxicity, and excellent biodegradability. Till now, mesoporous inorganic solids, such as zeolites [7,8] and silica [9,10] (pure or functionalized by a postsynthetic modification), biocompatible dendritic macromolecules or polymers [11], and lipids [12] and metal clusters [13,14], have been taken as possible candidates for drug storage. However, these materials suffer from several limitations [7], namely, low drug-loading capacities (<5 wt %), rapid drug release, and toxicity. As far as the dendritic macromolecules [8] are concerned, these drugs can be encapsulated efficiently, but it is hard to achieve controlled release of drugs because of the absence of a well-defined porosity [15,16]. Similarly, organic molecules grafted on pore walls enable inorganic porous solids to perform better and controlled release of drugs, but these organic polymers possess low drug-loading capacity [17,18]. So, the development of multifunctional drug carriers will be a research trend in the future.

The extensive class of crystalline materials (metal-organic frameworks, MOFs) [19,20] are composed of metal centers or inorganic clusters bridged by organic units through coordinate bonds. As a result, a wide variety of metal ions and organic linkers have been designed and selected to synthesize over 15,000 MOFs that have displayed excellent properties [21]. Compared to their organic (dendritic macromolecules) or inorganic counterparts (zeolites), MOFs can be functionalized to give ubiquitous ability, as the ligand-producing variety of MOFs can be postmodified, or alternatively, the ligand itself may possess special functional groups [22,23]. In addition to their diverse and versatile framework, MOFs also display extraordinary properties, such as high surface areas, ultrahigh porosity, and thermal stability [24,25], which make them a potential candidate for applications in areas such as gas storage and separation [26,27,28,29,30,31], catalysis [32,33], chemical sensors [34,35], drug delivery, and biomedical imaging [36,37,38,39,40,41]. In this regard, Yaghi et al. [42] pioneeringly synthesized a series of MOFs with an exceptionally high surface area (4500 m^2^·g^−1^) and extra-large pores capable of binding polycyclic organic guest molecules. The pore volume and surface area of MOFs are well beyond comparison to most porous crystalline zeolites and porous carbon. Recently, Horcajada et al. studied the absorption of ibuprofen by two rigid MOFs—MIL-100 and MIL-101—which exhibited very high drug storage capacity and possessed completely controlled drug release under physiological conditions in 3–6 days [43]. However, metals (Cr, Cu, Gd, etc.) or ligands of MOFs have a certain level of toxicity, which is a hurdle for their real-world utility. From the perspective of biological applications, researchers have designed and synthesized some biocompatible MOFs using bio-ligands and nontoxic metal ions. For example, Mantion et al. have reported the first example of a peptide-based MOF [44], which was constructed using oligovaline peptides as organic linkers to coordinate to Ca^2+^ centers. The first therapeutically active MOFs based on nontoxic iron (LD_50_ = 30 g·kg^−1^) and nicotinic acid were synthesized and characterized by Miller et al. [40]. As the therapeutic agent and a constituent of the framework, the nicotinic acid released rapidly by degradation of the hybrid phase. This paved a new pathway for the development of new drug delivery systems based on biocompatible metals and pharmaceutical molecules displaying complex functions, and it indicated that bio-ligands play an important role in bio-applications.

**Figure 1:**
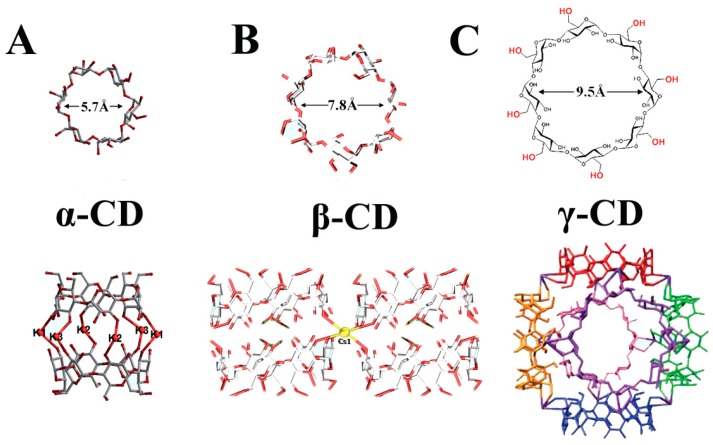
Structural representations of three types of cyclodextrins (CDs) and a schematic diagram of coordination between α/β/γ CDs and metal ions. (**A**) The [K_6_ (CD)_2_] dimer torus. (**B**) View of two pairs of β-CD molecules combined with the individual Cs^+^ ion; they have the reverse packing arrangement. (**C**) (γ-CD)_6_ cube of CD-MOFs. Reprinted with permission from [61,62,63,64], Copyright ACS, 2011; RSC, 2017; RSC, 2017; and RSC, 2016.

It is worth mentioning that cyclodextrins (CDs) play an irreplaceable role in the field of pharmaceutics and have received significant attention [45,46]. Cyclodextrin is a cyclic oligosaccharide that is produced in bulk by enzymatic action on starch. It exists in three forms, namely, α, β, and γ, which represent six, seven, or eight glucopyranose units, respectively (Figure 1) [47,48]. CD can combine with many inorganic or organic molecules to form host–guest inclusion complexes, which consist of a hydrophobic internal cavity and a hydrophilic external surface. For γ-CD, the aperture size can be up to 1.69 nm and is capable of encapsulating small drug molecules. Meanwhile, the hydroxyl group lying at the external surface of CD can provide hydrophobic binding sites to combine special groups to induce unique effects [49,50]. In view of these traits, many studies have shown that CDs and their derivatives as drug carriers or pharmaceutical formulations improve the stability, solubility, and bioavailability of drugs [51,52]. CD-based inclusion complexes or polymers have been widely investigated in drug-loading experiments, but they have certain drawbacks with inorganic/organic materials, such as low drug-loading capacity and burst release [53,54,55,56]. Inspired by these works, Smaldone et al. [57] reported the first example of a cubic CD-MOF-K (called CD-MOF-1) that was prepared in aqueous media at ambient temperature and pressure. Especially, CD-MOF was synthesized from edible building blocks of food-grade γ-CD with a salt substitute. Other CD-MOFs have been readily obtained using salts of Na^+^ and Fe^3+^ [58,59], which have developed into an extensive new class of CD-containing polymers. Interestingly, heteromorphic CDs and metal ions are capable of forming CD-MOFs having diversified structures. Figure 1 gives the structural representations of three types of CDs as well as the schematic diagram of the coordination associated between three types of CD and metal ions. Figure 1A indicates that two α-CDs are coordinated together to form a [K_6_ (CD)_2_] dimer torus in the secondary face to secondary face pattern, while, Cs^+^ ion are linked with two pairs of β-CDs and display a reverse packing arrangement (Figure 1B). In another case, eight-coordinate K^+^ ions are coordinated with γ-CD to produce an assembly of (γ-CD)_6_ cubes, where the six γ-CD units occupy the faces of a cube. These results demonstrated the diversity and controllability of CD-based MOFs which can meet requirements associated with various applications. The common feature of CD-MOFs is that their coordination frameworks have a high specific surface area (SSA), a new class of porosity, and the additional advantage of being edible. The study of CD-MOFs is still in its initial phase, although it is gaining popularity. So far, CD-MOFs have shown potential application in separation, sensing, and drug delivery [60]. In this review, we provide a comprehensive summary of the developments that have emerged over the past decade with respect to synthetic methods and applications of CD-MOFs. The advantages and disadvantages of different CD-MOFs, as well as CD-MOF-based drug delivery systems, are the focus. To conclude the review, we discuss the outlook on CD-MOFs and possible challenges in this field.

## 2. Synthetic Strategy of CD-MOFs

Based on the unique structural features of CD molecules, comprising a hydrophobic central cavity and a hydrophilic outer surface, they mainly coordinate with alkaline earth metals to produce new metal-organic frameworks (namely, CD-MOFs). The modulation and selection of CDs and alkaline metal ions leads to diverse structures in the resulting CD-MOFs. The main synthetic strategies used to develop CD-MOFs in the reported literature are summarized below.

### 2.1. Vapor Diffusion Method

The vapor diffusion method is an early synthetic pathway to develop MOFs. When compared to the conventional solvothermal method, the synthetic conditions for this method require ambient temperatures and pressures. This methodology is beneficial to generate crystals but requires solubility of ligands, which therefore limits its wide application for the synthesis of MOFs. A series of γ-CD-MOFs were synthesized and structurally characterized by Smaldone and co-workers for the first time, and then Forgan’s group prepared γ-CD-MOFs by combining γ-CD with K^+^, Rb^+^, and Cs^+^ in aqueous medium followed by vapor diffusion of MeOH. They obtained single crystals of γ-CD-MOFs within 2–7 days that had crystals sizes of around 200–400 µm [57,65]. To synthesize much smaller CD-MOF crystals, Furukawa et al. made modifications in the previous reports and synthesized pure, homogeneous, and monodisperse crystals of nano- and micro-γ-CD-MOFs by adding cetyltrimethy lammonium bromide (CTAB), which led to a crystal size of about 1–10 µm. Interestingly, they successfully synthesized the nano-γ-CD-MOFs by the addition of both CTAB and methanol [66]. Later, Liu et al. devised a fast and effective solvent evaporation approach to synthesize γ-CD-MOFs. By changing the reactant concentrations, temperatures, time, γ-CD ratio to KOH, and surfactant concentrations, they efficiently tuned the crystal dimensions of γ-CD-MOFs. Notably, the synthetic time for γ-CD-MOFs was greatly reduced to 6 h [67]. Subsequently, Stoddart’s group initially utilized α-CD and Rb^+^ to coordinate α-CD-MOF in aqueous solution [68]. This method was a pioneering step in the synthesis of α-CD-MOFs and offered a new way to synthesize CD-MOFs. Sha et al. employed α-CD, KOH, and NaOH to prepare two α-CD-MOFs using the same methodology [64,69]. Using the same procedure, α-CD-MOF-K and β-CD-MOF-K/γ-CD-MOF-K polymers were also prepared by Li and co-workers. These investigations have further expanded, which enriched the family of CD-MOFs and induced researchers to explore additional applications of CD-MOFs [70]. Recently, Qiu et al. proposed a facile and green seed-mediated method to rapidly synthesize γ-CD-MOFs [71]. The results showed that the crystal structure was not changed, while the size of the MOF was observed to decrease when the seed was added. These strategies provide a facile and green technique in synthetic methods of CD-MOFs.

### 2.2. Hydrothermal/Solvothermal Method

In the hydro/solvothermal method, the reaction takes place in the mother liquor under high-temperature and high-pressure conditions. Like classical synthetic protocols, this method has been widely employed in the synthesis of MOFs. Several parameters, such as the reaction time, temperature, stoichiometry, dilution pH, and additives, play important roles in the synthetic process. According to the unique traits of CDs, a new β-CD-MOF was successfully prepared from β-CD and Na_2_C_2_O_4_ by adding a solution mixture of methanol and water and heating it at 160 °C for 3 days. This represents the first example of chiral helices constructed using β-CD molecules and metal ions. Forgan et al. loaded the drug 5-FU over β-CD-MOF, and the subsequent results indicated that the β-CD-MOF could become a potential drug carrier [59]. Subsequently, this group also synthesized some other β-CD-MOFs by changing the temperature, molar ratio, and solvent mixture ratio by following a similar method. Sha et al. obtained a new type of CD-MOF by the solvothermal method using α-CD and KOH. The α-CD-MOF possessed a chiral helical chain which had also been used to load 5-FU [64]. Liu et al. proposed and synthesized a novel template-induced approach to synthesize CD-MOFs. They selected 1,2,3-triazole-4,5-dicarboxylic acid (H_3_tzdc), methyl benzene sulfonic acid (TsOH), or ibuprofen molecule (IBU) as templating agents to synthesize different Cs-β-CD-MOFs under hydro/solvothermal condition. The manner in which the templating agents changed the crystallinity and porosity of Cs-β-CD-MOFs was also confirmed by the corresponding investigation of the quantity of control experiments. Importantly, this was the first time that the template-induced approach was employed for the synthesis of β-CD-MOFs with the hydro/solvothermal method [63]. Two chiral lead metal-organic nanotubes were synthesized by Wei et al. [72]. It is worth mentioning that they were built by β- or γ-CD and Pb^2+^ through a biphasic solvothermal reaction. This was the first time ever that any group had used metal ions to synthesize CD-MOFs other than alkali-earth metals.

### 2.3. Microwave-Assisted and Conventional Methods

**Figure 2:**
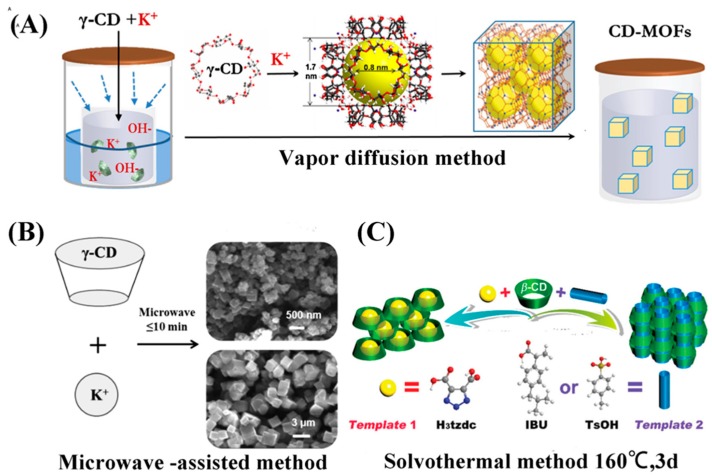
The three synthetic routes of CD-MOFs. (**A**) the schematic representation of γ-CD-MOF synthesis by vapor diffusion method, (**B**) the different sizes of γ-CD-MOF were adjusted under the microwave method, (**C**) the schematic view of different structural formation of CD-MOF by using different templates in the reaction process. Reprinted with permission from [63,78,80], copyright RSC, 2017; ACS, 2017; and Elsevier, 2017.

The microwave-assisted method is a developing chemical synthetic path and has been successfully employed for the synthesis of various materials such as metals, metal oxides, inorganic hybrids, MOFs, etc. This synthetic method offers the advantages of simple, rapid, inexpensive, relatively green, and efficient nonconventional heating and high yield. Li et al. synthesized MIL-100-Cr by the microwave technique for the first time [73]. Subsequently, using this superior method, other MOFs, namely, MOF-5 and MIL-53-Fe, were also synthesized [74,75,76,77]. Liu et al. also obtained γ-CD-MOFs using the microwave-assisted method in merely 1 h. In order to obtain micro- and nanometer-sized crystals, they systematically investigated the size and morphology of γ-CD-MOF by altering the reaction time, temperature, and solvent ratio [78]. Sha et al. also synthesized a β-CD-MOF using the conventional method. In this conventional method, β-cyclodextrin and NaOH were dissolved in deionized water and C_2_H_5_OH, and colorless crystals were gradually generated in two weeks [79]. The procedure of the conventional method is similar to vapor diffusion, but it only needs moderate conditions. However, this process is time-consuming and the crystal size is difficult to control. The various synthetic strategies adopted to synthesize a variety of CD-MOFs and their applications are summarized in Table 1. The illustrations of all the three synthetic methods are presented in Figure 2. All the above mentioned synthetic methods have their own advantages. The reports indicate that factors such as reactant concentrations, temperatures, time, molar ratio, and solvents affect the sizes or dimensions of CD-MOFs.

### 2.4. Postmodification Method

Since the structure of CD can be modified, it is therefore important to improve the stability of CD-MOFs in an aqueous medium as well as in the physiological environment. So, researchers have been exploring the possibilities of modification in CD-MOF structures to improve their range of applications. Firstly, nano- and micro-sized cubic gel particles from CD-MOFs were prepared by Sada et al. using a bottom-up approach. They utilized two epoxy groups of ethylene glycol diglycidyl ether to cross-link the adjacent hydroxyl groups of each γ-CD in the CD-MOF pores [66]. This modification not only improved the stability of the CD-MOF but also controlled the sizes and shapes of the resulting MOFs. This modification to CD-MOFs further facilitated their application as potential drug carriers and cell-support materials. Following this approach, polymerization of 3,4-ethylenedioxythiophene (EDOT) in CD-MOF was also investigated [81]. The investigations indicated that CD-MOF@EDOT became thermally stable and exhibited preferable solubility in water. Subsequently, poly(acrylic acid), hydrophobic C_60_, and cholesterol as a functional group were incorporated with CD-MOFs by using a facile procedure [82,83,84], which also improved the stability of CD-MOFs in water and enhanced their drug-loading capacities. The postmodification strategy is used to graft functional groups to CD-MOFs, offering a new platform to expand the applications of CD-MOFs.

### 2.5. The Stability of CD-MOFs

**Figure 3:**
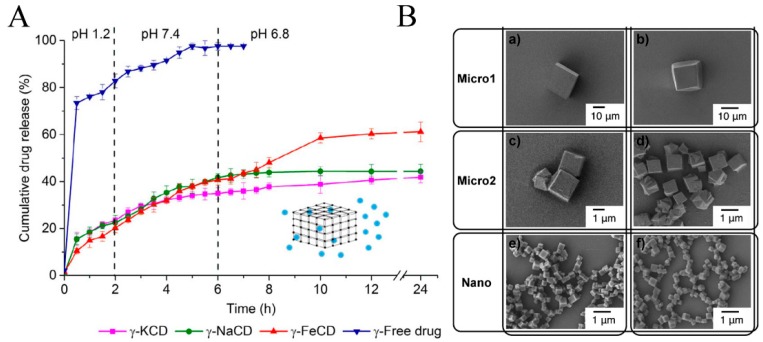
The drug release curve of three CD-MOFs in different pH solutions (**A**) and morphologies of CD-MOFs in different sizes (**B**). Reprinted with permission from [58,66], copyright Elsevier, 2018 and Wiley, 2012.

The structural stability of MOFs in bio-applications have been discussed and studied by researchers due to the weak bond between organic linkers and metal ions in CD-MOFs. Fortunately, CD-MOFs have a certain stability and solubility because of their hydrophobic internal cavity and hydrophilic external surface in aqueous media. Firstly, the powder X-ray diffraction for loaded drug CD-MOFs were found to coincide over free CD-MOFs in the report, which demonstrated its structural stability [58,83,88]. Meanwhile, the drug release curve of drug-loaded CD-MOFs showed no obvious burst release effect compared to the control group (Figure 3A). This phenomenon is mainly attributed to the fact that the framework gets slowly collapsed to release the drug by the cleavage of weak bonds between drugs and functional groups of the framework or drug [38,89]. Secondly, the size and morphology of CD-MOFs also affect their stability. Results indicated micro/nano-sized CD-MOFs maintained their integral morphology and porosity (Figure 3B). TGA curves for CD-MOFs did not show significant weight loss up to 200 °C, indicating that the pores were fully vacated but the framework remained thermally stable [57,83]. Integrating other functional groups or materials with CD-MOFs’ surface can improve their stability in water or their physiological environment, which is analogous to the postmodification method. This strategy does not only improve the stability of CD-MOFs and make them multifunctional carriers, but it also supports a way to extend their application.

## 3. Encapsulation Technology of CD-MOFs

Encapsulating molecules into CD-MOFs could improve their properties and broaden their scope of application. Therefore, encapsulation technologies of biocompatible CD−MOFs have been explored theoretically and experimentally so that the prospects of CD-MOFs in research areas such as food, pharmaceuticals, and packaging can be explored. The various factors and conditions regarding the different encapsulation techniques are discussed in this section of review. The comparison of advantages and disadvantages associated with various encapsulation methods, as well as current research trends, are also discussed.

### 3.1. Absorption Method

Absorption encapsulation, also known as impregnation encapsulation, is the most common method that generally involves three steps: (i) the synthesized CD-MOFs are immersed in solvents or rinsed with solvents and thereafter dried to obtain the activated CD-MOFs; (ii) guest molecules are dissolved in suitable solvents or they are filled into a confined space containing CD-MOFs; (iii) the drug molecules are encapsulated into the activated CD-MOFs. Lv et al. investigated the encapsulation of sucralose into γ-CD-MOF (K^+^) [90]. The results indicated that the thermal stability of encapsulated sucralose in the solution was dramatically improved, which was led by a CD-MOF as a shell playing a protective function, with weak interactions between functional groups of sucralose and the CD-MOF. For the sake of comparison, neutral CD-MOFs were converted into alkaline CD-MOFs which were more effective to protect sucralose. The variable protecting properties of the same CD-MOFs at different pH levels may be ascribed to the fast hydrolysis of sucralose in an alkaline environment compared to a neutral environment. The stability of encapsulated curcumin in curcumin @ CD-MOFs was also enhanced at least up to three orders of magnitude without altering the crystallinity of the CD-MOFs [91]. This resulted in a hydrogen bond interaction between the OH group of the cyclodextrin moiety of CD-MOFs and the phenolic hydroxyl group of curcumin, such that curcumins in the pores of CD-MOFs were more stable. Hartlieb et al. found that the uptake of ibuprofen into CD-MOFs was low when relatively nonpolar solvents were used [88]. When ethanol was used as a solvent, the loading of ibuprofen into CD-MOF increased substantially to 26 wt %, which was close to the theoretical value calculated by Monte Carlo simulations. They proposed that the mechanism of encapsulation is related to the anion exchange process, where the ibuprofen is deprotonated by the hydroxyl group present in CD-MOFs, and this newly formed anion now balances the positive charge of the framework. The above examples demonstrate that the solvent plays a critical role in controlling the efficiency of encapsulation in CD-MOFs. In addition, the porous structure of CD-MOFs also affects the encapsulation efficiency. Al-Ghamdi et al. reported two different γ-CD-MOF-Ks using potassium hydroxide and potassium benzoate as sources, called CD-MOF-a and CD-MOF-b, respectively [86]. The two K-CD-MOFs possessed different types of pores, which were suitable candidates to encapsulate acetaldehyde. Results of encapsulation indicated that the aldol condensation reaction operated during encapsulation of acetaldehyde into CD-MOF-b. These results also indicate that acetaldehyde can successfully be encapsulated into the CD-MOF-b. Thus, CD-MOF-b has the potential to encapsulate volatile organic compounds.

In general, the adsorption encapsulation method is convenient and widely used. It is noteworthy that the mass ratio between the drug molecules, the nature of the encapsulation materials, and encapsulation time are critical. For example, Sun et al. evaluated the effect of encapsulation time on the encapsulation of 5-FU by Na-CD-MOF with a mass ratio of (Na-CD-MOF:5-FU = 1:1–1:7) [86]. The results showed that the encapsulation ratio was improved with increasing the mass ratio of 5-FU and Na-CD-MOF. When the mass ratio was 1:5 for Na-CD-MOF to 5-FU, then encapsulation was optimum. At the same time, when the encapsulation time was extended, the encapsulation efficiency also increased. After 48 h, however, drug loading was observed to decrease. This observation indicated that the partial desorption of 5-FU adsorbed on the surface of CD-MOF occurred when soaking time was increased. Also, the encapsulation temperatures can affect drug-loading efficiency [87]. It has been found that temperatures of 20, 25, 30, and 37 °C are related to encapsulation efficiency, and thereby, they establish a new approach to optimize drug encapsulation.

### 3.2. Grinding Method

Guest molecules can be successively encapsulated into CD-MOFs through mechanical grinding. Typical procedures are as follows: Guest molecules and CD-MOFs are accurately weighted in stoichiometric ratio. Both the compounds are thereafter grinded using suitable solvents for a period of time at a designated temperature. The resulting products are rinsed using a certain amount of solvents and desiccated until a constant weight is obtained. 

Some other factors also affect the inclusion effect, such as molecular ratio, inclusion temperature, and inclusion time, which have been tested by Yang’s team. In 2015, they reported a new CD-MOF based on β-CD, which represented the first example of chiral helices constructed by β-CD molecules and metal ions. In this work, 5-FU was taken as a model drug molecule to investigate the inclusion performance of a CD-MOF at room temperature. The result showed that the inclusion rate for the CD-MOF was 23.02% when the 5-FU-to-CD-MOF-Na molar ratio was 1:1, which was higher than that of β-CD (15.73%) [59]. After that, to obtain the optimal encapsulation conditions, they further explored the influence of various factors by the orthogonal design of four factors and three levels. The drugs azithromycin (AM) and ferulic acid (FA) were selected as the main components, and AM/K/β-CD/MOF and FA/K/β-CD/MOF were prepared by the grinding method [85,92]. The encapsulation efficiency was improved by the orthogonal test regarding the mole ratio of the main guest molecule-inclusion material, grinding time, dropping time, and inclusion temperature as factors and the inclusion rate as the index. The result showed that the best inclusion condition of AM was when the mole ratio of drug to K-CD-MOF was 1:1 during grinding, the inclusion time was 2 h, and the inclusion temperature was 40 °C. In the mentioned reaction, the inclusion rate was highest, and the average rate of inclusion was 33.91 ± 1.2%. However, the maximum encapsulation rate of FA was observed when the mole ratio of FA to K/β/CD-MOF was 3:1, the dropping time was 60 min, and the inclusion temperature was 40 °C. Furthermore, after careful analysis of variance, they concluded that the order of different factors on the inclusion rate was: inclusion temperature > the mole ratio of drug to CD-MOFs > grinding time > dropping time.

### 3.3. Cocrystallization Method

Cocrystallization is one of the emerging crystal engineering techniques for modulating pharmaceutical performance. It had been subdivided into three categories by Sun, namely, separate solvent-mediated cocrystallization, solid-state cocrystallization, and high-throughput cocrystallization [92]. Amongst the three cocrystallization methods, solvent-mediated cocrystallization is widely used for the encapsulation of CD-MOFs. Michida et al. studied the crystallization of CD-MOFs with inclusion of FA at the molar ratio of γ-CD:KOH:FA of 1:8:2 and 1:8:8, respectively [93]. In order to ascertain the synthetic conditions of FA/CD-MOF, they first investigated the effect of pH values from 12.7 to 13.6 when preparing CD-MOF. The pH value of the mixed solution was 13.1, which was suitable for the crystallization of pure CD-MOF. Unfortunately, the molar ratio of FA to γ-CD in FA/CD-MOF prepared using the above prerequisite was only 0.15. When the molar ratio was altered to 1:8:8, the molar ratio of FA to γ-CD was up to 1.06. These results indicated that FA may get encapsulated into γ-CD and also adsorbed on the pore wall in the (γ-CD)_6_ cube. In this case, these salts seem to facilitate FA/CD-MOF crystal growth despite the fact that they had not produced aqueous solutions of sufficient basicity to deprotonate the hydroxyl group of γ-CD. To grow reliable cocrystals, much attention should be paid to controlling the stoichiometric compositions of the crystals. 

Although the outcomes of the cocrystallization processes from a solution are not always predictable, we firmly believe that the future of encapsulation technology research will significantly benefit from the optimization of solvent-mediated cocrystallization. This is a prevailing and promising approach in preparing commercial-scale cocrystals because of the availability of solution crystallization equipment in pharmaceutical manufacturing plants.

According to the literature, the encapsulation purposes of CD-MOFs as a host mainly improved the solubility and stability of many drugs, thereby improving bioavailability in practical applications. Based on the above three encapsulation technologies of CD-MOFs, they all have their own advantages, and which encapsulation technology is chosen is mainly determined by the physicochemical properties of CD-MOFs and guest molecules, such as polarities, sizes, functional groups, and thermal stability. Certainly, adopting different methods can result indifferent encapsulation effects. We briefly summarize the commonality and individuality of applicable conditions and influencing factors under encapsulation drugs and offer some suggestions about how better choose drugs and encapsulation methods to obtain better encapsulation results. First, drug molecules should have certain functional groups to facilitate bonding with the wall of CD-MOFs’ surface to obtain a relatively high encapsulation load and improve its stability in the CD-MOF cave. Second, the size and length of drug molecules should be considered under the encapsulation process because the aperture size of CD-MOFs is restricted. Similarly, excessive size and length of drug molecules can produce a steric hindrance leading to low drug capacity. Further, drugs should have some defects in solubility and stability at ambient temperatures and pressures. The appropriate polarity and small size of a drug play important roles in adsorption encapsulation because drug molecules are prone to enter into holes and combine with CD-MOFs. CD-MOFs and nonpolar drugs retain their structural stability under the grinding process, and β-CD-MOFs are often used as hosts in grinding encapsulation because they have a unique chiral helix structure and relative stability of coordination between β-CD molecules and metal ions. As the condition of cocrystal encapsulation is increasingly strict, the drug should be stable and soluble in an alkaline solution because the synthetic solution pH of CD-MOFs is highly basic, which may be limit application. In general, the three methods have their own merits, but adsorption encapsulation is widely used because of its convenience, universality, and adjustment.

## 4. Drug Delivery

Many inexpensive and green CD-MOFs based on α-, β-, γ-CDs, and metal ions have been expected as potential materials for drug delivery [60]. In addition, some advances in adsorption and enrichment of drugs or biomolecules in biological samples have also been achieved using CD-MOFs. However, these fields have not been deeply explored due to their short history. In this regard, only a few representative examples are introduced.

Some drug molecules have poor physicochemical and biopharmaceutical properties which limit their formulation development. To overcome the intrinsic limitations of these therapeutic molecules and to ensure controllable drug delivery, great efforts have been made to explore suitable drug carriers. Systems based on CD-MOF complexation camouflage the undesirable properties of drugs and lead to synergistic or additive effects. This would possibly resolve the challenges in the field of pharmaceutics related to the development and commercialization of therapeutic agents.

**Figure 4:**
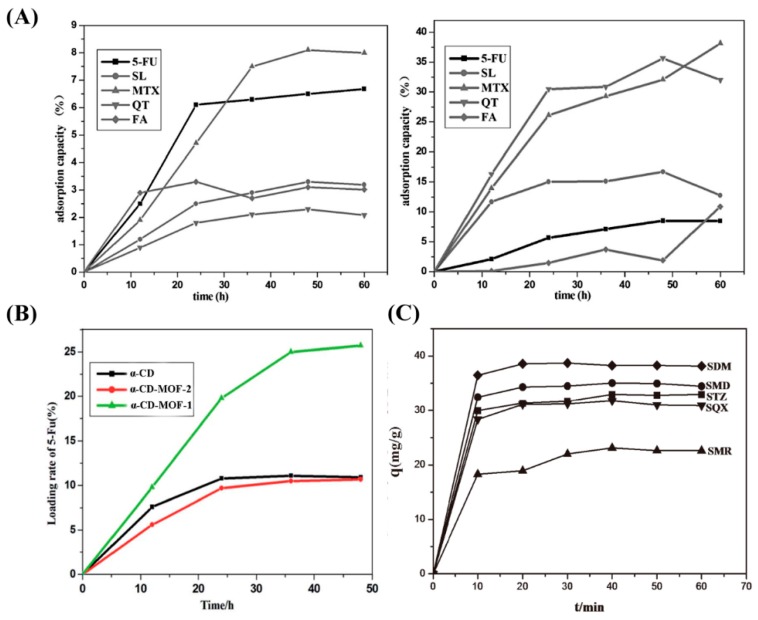
Time dependence of five drug adsorption capacities in α-CD (left) and Na-α-CD-MOF (right) (**A**), 5-Fu drug-loading rate in α-CD and two α-CD-MOFs (**B**), and four sulfonamide drug adsorption rates in γ-CD-MOF (**C**). Reprinted with permission from [64,69,99], copyright RSC, 2016; Elsevier, 2017; and Elsevier, 2018.

**Figure 5:**
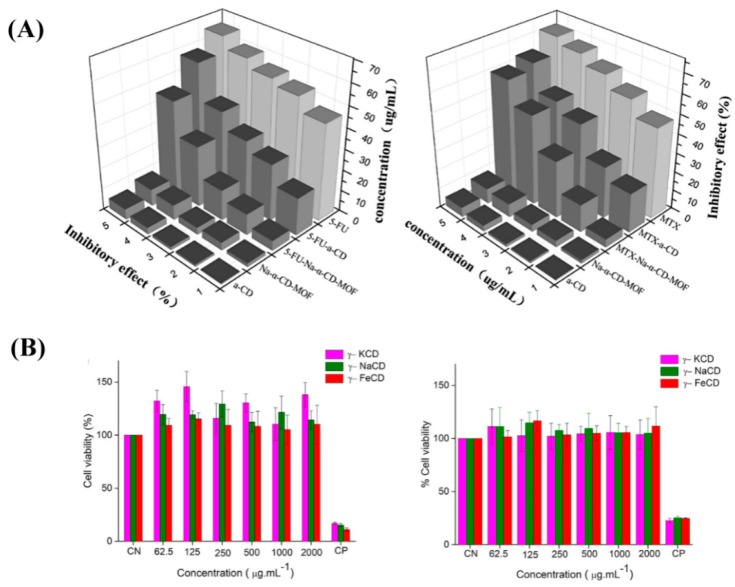
(**A**) Chart of cytotoxicity on HepG2 cells for α-CD, Na-α-CD-MOF, 5-FU (left), MTX (right), 5-FU-α-CD (left), MTX-α-CD (right), 5-FU-Na-α-CD-MOF (left), and MTX-Na-α-CDMOF (right). (**B**) Cytotoxic effect of three γ-CD-MOFs at a concentration of 62.5–2000 μg/mL: (left) HepG2 cell lines and (right) Caco-2 cell lines. Reprinted with permission from [58,69], copyright Elsevier, 2018 and Elsevier, 2017.

**Figure 6:**
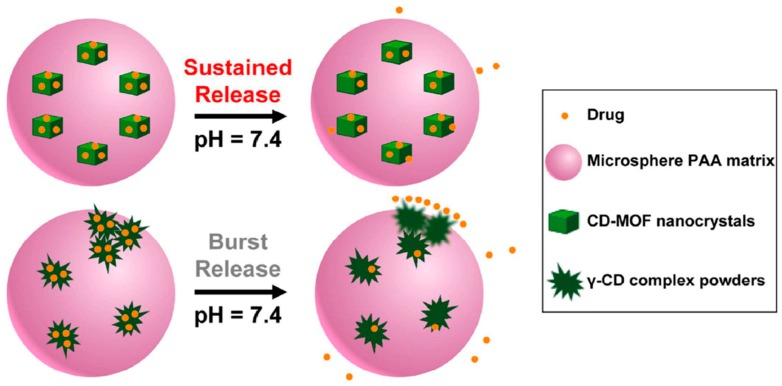
Illustration of burst and sustained drug release mechanisms of γ-CD complexes and CD-MOFs within PAA matrix. Reprinted with permission from [84], Copyright RSC, 2017.

**Figure 7:**
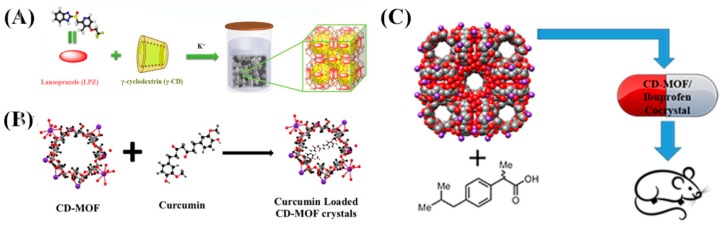
Illustration of CD-MOFs loading drugs. (**A**) Lansoprazole, (**B**) curcumin, and (**C**) ibuprofen loaded by CD-MOFs. Reprinted with permission from [80,88,91], copyright Elsevier, 2017; ACS, 2017; Elsevier, 2016.

Drug molecules are loaded onto CD-MOFs through the principle of host–guest molecular inclusion, which is identical to CDs. Nowadays, the CD-based pharmaceutical product is a well-developed field with many commercial examples, such as prostaglandin E2/βCD (Prostarmon E™ sublingual tablets) and itraconazole/2-hydroxypropyl-β-CD oral solution (Sporanox®) [94,95]. For comparison, Sha et al. investigated drug adsorption by Na-α-CD-MOF and α-CD, as shown in Figure 4A and Table 2. The results show that Na-α-CD-MOF possesses remarkable drug adsorption for all the model drugs compared to the α-CD matrix. Research on the cytotoxicity of Na-a-CD-MOF exhibited amazing results, showing that the loaded drug-Na-a-CD-MOFs showed lower cytotoxicity than that of the CD-matrix under the same drug concentration (Figure 5A). However, the solubility and biocompatibility of the drugs in Na-α-CD-MOFs were not ideal, perhaps due to the weak noncovalent interactions between the building blocks and metal centers of Na-a-CD-MOFs. Thus, the lower cytotoxicity indicates that Na-α-CD-MOFs possess very attractive prospect in drug delivery. Meanwhile, this research group synthesized two novel K-α-CD-MOFs and performed a loading drug experiment. The K-α-CD-MOF-1 displayed remarkable 5-FU adsorption in comparison to that of the α-CD matrix due to its larger cavity size (Figure 4B) [64]. The study of ibuprofen incorporated in CD-MOF-1 in pharmaceutically relevant quantities was performed by Hartlieb et al. using in vivo and in vitro experiments [88]. In vitro viability studies revealed that the CD-MOF has very little effect on the viability of the cells value when the concentration was 100 μM (Figure 5B). From these studies, they concluded that, following oral administration, CD-MOF-1 cocrystals of ibuprofen exhibited similar bioavailability and rapid uptake in blood plasma. In addition, the cocrystal had the added benefit of a 100% longer half-life in blood plasma samples than the pure salt form (Table 3). Combined with Monte Carlo simulations and experiments, the high loading capacity and biocompatibility of CD-MOF-1 was attributed to electrostatic interaction. The protonated ibuprofen formed anions to balance the positive charge of the framework to improve the solubility and efficacy of the drug. It is clear that CD-MOF-1 is effective as a delivery vehicle. Two new CD-MOFs (NaOH(C_42_H_70_O_35_)·9H_2_O (**A**) and KOH(C_42_H_70_O_35_)·9H_2_O(**B**)), based on natural β-CD and alkali metals (Na^+^/K^+^), were synthesized and characterized by Sha’s group [79]. The characterization results revealed that compounds **A** and **B** possessed bowl-like pores and the “8”-type double channel configuration. Two kinds of drug molecules (5-FU and quercetin) with different structures and sizes were successfully incorporated into the compounds at the same time. This study verified that **A** and **B** could be new types of multifunctional drug carriers and also offered the possibility of synergistic drug delivery based on CD-MOFs. Recently, Jin et al. designed and synthesized nano- and micro-sized γ-CD-MOFs based on a novel approach with controlled nucleation and growth, which could conveniently and quickly obtain uniform CD-MOFs and keep their structural integrity [96]. Through loading experiments with resveratrol, CD-MOFs exhibited a relatively high loading capacity and a consequently sustained release, indicating that they could be used as delivery carriers for drugs and controlled-release systems. Therefore, CD-MOFs show many advantages for drug delivery compared to traditional drug carriers. However, the use of CD-MOFs in the biomedical field is limited because of their physical frailness and high solubility in aqueous media, which may lead to their extended frameworks collapsing before reaching the target tissues or organs. To overcome this problem, surface modifications of CD-MOFs were investigated by researchers through grafting specially functional groups. Firstly, Li’s research group utilized hydrophobic C_60_ to incorporate γ-CD-MOFs in order to improve their stability in an aqueous environment without affecting the structural integrity and Brunauer–Emmett–Teller (BET) surface area. Thereafter, the new carrier system successfully loaded DOX and the resulting material showed a slow release effect in phosphate buffer saline [83]. Meanwhile, Zhang et al. also successfully modified cholesterol with the surface of CD-MOFs by using the same idea. The resultant product not only showed good tolerance in vivo but also increased the blood half-life of DOX up to four times [97]. In order to achieve greater stability and sustained drug release for CD-MOFs, Li et al. embedded CD-MOF nanocrystals into a biocompatible polymer (polyacrylic acid, PAA) matrix [84] to obtain a composite microspheres. The composite microspheres not only sustained drug release over a prolonged period of time but also showed lower cell toxicity (Figure 6). An efficient and pharmaceutically acceptable CD-MOF-based carrier for sustained drug release was prepared in this work. Furthermore, examples of other drugs encapsulated by CD-MOFs are shown in Figure 7, which also demonstrates that CD-MOFs are a promising drug carrier. It is interesting that water-soluble tetrakis (4-hydroxyphenyl) porphyrin (TCPP)-isolated C_60_ were encapsulated into the pores of CD-MOFs, leading to them showing fluorescent properties with CD-MOFs [98]. This work primarily introduced fluorescent properties into CD-MOFs, which offered a novel idea to exploit fluorescence imaging for CD-MOFs in vivo, but it was noted that the size and stability of TCPP/CD-MOFs could be controlled in the experiment. CD-MOFs not only inherited CD’s good biocompatibility in medical applications but also had a regular pore structure that was beneficial to loading drugs. The choice of drug is similar to encapsulation conditions. CD-MOFs as single-functional drug carriers are not enough to face complex physiological conditions and diseases. The multifunctional drug carrier is an inevitable trend of development. Incorporating biocompatible polymer matrices or functional groups to the surface of CD-MOFs would overcome the problems of rapid hydration in water and sustained release. The above studies have initiated the work on multifunctional CD-MOF carriers, and the next stage is integrating diagnosis and treatment, such as by combining targeted groups, fluorescein, and other drugs by structural modifications of CD-MOFs, leading to targeted therapy, imaging, and synergistic effects.

## 5. Other Bio-Applications

Due to the larger pore, poly hydroxyl groups, and stability of CD-MOFs, they were also applied to adsorb, separate, and quantify drugs or biomolecules to extend their bio-applications. These studies are briefly described and discussed below.

CD-MOFs have unique advantages in enriching and detecting drugs or biomolecules of biological samples, exhibiting high sensitivity and detection efficiency. Following this idea, Li et al. utilized γ-CD-MOFs to adsorb sulfonamides (SAs), reaching an enhancement effect before their High Performance Liquid Chromatography (HPLC) determination [99]. They also showed a high adsorption capacity and quickly bound the SAs due to hydrogen bonding interactions between SAs and the hydroxyl groups of CD-MOFs on the surface and cyclodextrin inclusion interactions. Adsorption rates of SAs are shown in Figure 4C. Meanwhile, the utility of CD-MOFs for adsorption and separation of drugs was demonstrated by Zhang et al. [106]. Ketoprofen, fenbufen, and diazepam were selected as model drugs, and they investigated the correlation of adsorption behavior in the γ-CD-MOF column under different separation conditions. The results indicated that the solvent and temperature were key factors that could affect retention and loading efficiency. For example, the adsorption capacity of ketoprofen and fenbufen increased when the hexane content increased, but the retention time and adsorption uptake of the three drugs showed different tendencies at different temperatures. Zou et al. designed and synthesized a novel LCD-MOF by γ-CD-MOF cross-linked to ethylene glycol diglycidyl ether and investigated it to enrich glycopeptides from biological samples. Due to the hydrophilic interactions of glycopetide and LCD-MOF, the enrichment result showed superior selectivity, high detection sensitivity, and reproducibility [107]. Recently, Kanamoto et al., for the first time, utilized CD-MOF-1 to encapsulate coenzyme Q10 [108]. The experiments indicated that CD-MOFs are not only capable of loading drugs but they can also encapsulate bio-enzymes by preserving their biological activity [108]. The report is an interesting work and will expand the scope of CD-MOFs in medical applications. Based on the above considerations, CD-MOFs may be a promising material for adsorption, separation, and purification of drugs or biomolecules in the pretreatment of biological samples.

## 6. Conclusions and Outlooks

In the presented review, the synthetic methods and applications associated with CD-MOFs were summarized and discussed. More importantly, attempts have been made to emphasize encapsulation technologies and drug delivery by CD-MOFs. In accordance with the physical properties and structural characteristics of host (CD-MOFs) and guest molecules, optimum inclusion conditions or encapsulating mechanisms were obtained by employing various encapsulation strategies or encapsulating the same or different molecules. These results indicated how quickly and efficiently a CD-MOF can encapsulate drug molecules, thereby improving its drug action. The drug loading of CD-MOFs is perceptibly higher than that of pure CDs, and the important point is that CD-MOFs have the ability to overcome a drug’s poor physicochemical and biopharmaceutical properties and can exhibit a controlled release effect in vitro. The molecules within the pores of CD-MOFs are prone to release early in an aqueous environment due to their hydrophilic external surface, which poses problems for the sustained and controlled release effect as well as the pharmacological effect in biomedicine. Hence, pertinent suggestions have been discussed in this review, for example: (i) in the synthetic aspect, how to synthesize other metal-based CD-MOFs (Fe^3+^, Mg^2+^, Ca^2+^, etc.), and how to optimize the synthetic procedure to improve and control the size of CD-MOFs; (ii) in postmodification, functional groups can be covalently or noncovalently bonded to linkers of the CD-MOF surface, which can improve the stability of CD-MOFs in an aqueous environment using a similar method. Although there are recent exciting research advancements in the area of CD-MOFs, there are still many challenges because the biomedical application of CD-MOFs is only in its infancy. Based on the above suggestions, we hope that CD-MOFs can become a multifunctional carrier so that it can quickly and efficiently load drugs for transport in the biomedical field. Of course, realizing those goals requires joint effort from different areas of experts, and these concerted efforts will contribute to promote the development of CD-MOFs and their applications in medicine, materials, and novel device fabrication.

## Figures and Tables

**Table 1:** Examples of CD-based metal-organic frameworks (MOFs).

CD-MOF	CD	Metal Ion	Molecular Formula	Metal Salt	Synthetic Method	Application	Reference
α-CD-MOF	α-CD	Na^+^	[Na(H_2_O)(C_36_H_60_O_30_)]·H_2_O	NaOH	Vapor diffusion method	Drug loading	[69]
		K^+^	K_3_C_72_O_67_H_134_/C_36_O_31_H_62_	KOH	Vapor diffusion/solvothermal method	Drug loading	[64]
			~	KOH	Vapor diffusion method	Formaldehyde Adsorption	[70]
		Rb^+^	Rb_5_(C_144_H_204_O_122_)_2_(H_2_O)	RbOH	Vapor diffusion method	Chiral separation	[68]
β-CD-MOF	β-CD	Na^+^	(C_42_O_35_H_7_0)_2_(NaOH)_4_·H_2_O	Na_2_C_2_O_4_	Solvothermal method	Drug loading	[59]
			Na(C_42_H_70_O_35_)·OH·9H_2_O	NaOH	Vapor diffusion method	Drug loading	[79]
		K^+^	K(C_42_H_64_O_35_)·11H_2_O	KOH	Solvothermal method	Drug loading	[85]
			K(C_42_H_70_O_35_)·OH·9H_2_O	KOH	Vapor diffusion method	Drug loading	[79]
		Cs^+^	Cs(OH)·(C_42_H_70_O_35_)	CsCl	Solvothermal method	Drug loading	[63]
			[Cs_1.5_(C_42_H_66.5_O_35_)]_2_	CsCl	Solvothermal method	Drug loading	[63]
		Pb^2+^	[Pb_14_(β-CD)_2_]·3C_6_H_12_O·35H_2_O	PbCl_2_	Solvothermal method	I_2_ absorption	[72]
γ-CD-MOF	γ-CD	K^+^	[(C_48_H_80_O_40_)(KOH)_2_(H_2_O)_8_·(CH_3_OH)_8_]_n_	KOH	Vapor diffusion method	Dye/gasabsorption	[57]
			~	K_2_CO_3_	Vapor diffusion method	~	[57]
			~	KF	Vapor diffusion method	~	[57]
			~	K_2_(azobenzene-4,4″-dicarboxylate)	Vapor diffusion method	~	[57]
			~	KCl	Vapor diffusion method	~	[57]
			~	KBr	Vapor diffusion method	~	[57]
			K_4_(C_96_H_160_O_80_)(C_7_H_5_O_2_)_2_(OH)	C_7_H_5_KO_2_	Vapor diffusion method	Acetaldehyde Adsorption	[86]
		Rb^+^	[(C_48_H_80_O_40_)(RbOH)_2_(H_2_O)_11_·(CH_3_OH)_2_]_n_	RbOH	Vapor diffusion method	CO_2_absorption	[61]
			[(C_48_H_81_O_39_N)(RbOH)_2_(H_2_O)_8_]n	RbOH	vapor diffusion method	CO_2_absorption	[62]
		Cs^+^	Cs_2_(C_48_H_80_O_40_)(OH)_2_	CsOH·H_2_O	Vapor diffusion method	Gasabsorption	[65]
			C_24_H_40_CsO_20_·OH·CH_3_OH	CsOH·H_2_O	Vapor diffusion method	Gasabsorption	[65]
		Na^+^	C_96_H_166_O_83_Na_2_·2(OH)·CH_3_OH·4(H_2_O)	NaOH	Vapor diffusion method	Gasabsorption	[57]
			~	NaOH	Vapor diffusion method	Drug loading	[58]
			~	Na_2_CO_3_	Vapor diffusion method	~	[57]
			~	NaBPh_4_	Vapor diffusion method	~	[57]
		Sr^+^	C_48_H_80_O_40_SrBr_2_·3(H_2_O)	SrBr_2_	Vapor diffusion method	Gasabsorption	[65]
		Li^+^	K_1.23_Li_0.77_(C_48_H_80_O_40_)	LiOH·H_2_O	Vapor diffusion method	CO_2_absorption	[87]
		Pb^2+^	[Pb_16_(γ-CD)_2_]·14H_2_O	PbCl_2_	Solvothermal method	I_2_ absorption	[72]
		Fe^3+^	~	FeCl_3_	Vapor diffusion method	Drug loading	[58]

**Table 2:** Overview of drug encapsulation in classical MOF particles and CD-MOFs.

MOFs	Metal Ion	Organic Linker	Drug	Loading Method	Rate (wt %)	Reference
MIL-53(Fe)	Fe^3+^	1,4-benzene dicarboxylic acid	Ibuprofen	Impregnation method	20	[100]
MIL-101(Cr)	Cr^3+^	1,3,5-benzenetricarboxylic acid	Ibuprofen	Impregnation method	20	[43]
BioMIL-1	Fe^3+^	nicotinic acid	Nicotinic acid	Impregnation method	75	[40]
MIL-88A(Fe)	Fe^3+^	nicotinic acid	Busulfan	Impregnation method	8 ± 1	
ZIF-8	Zn^2+^	2-Methylimidazole	Doxorubicin	Impregnation method	4.9	[101]
UiO-66-2-NH_2_	Zr^4+^	2-aminoterephthalic acid	5-FU	Impregnation method	27	[102]
γ-CD-MOF	K^+^	γ-CD	Lansoprazole	Cocrystallization method	23.2 ± 2.1	[80]
	K^+^(CTAB)	γ-CD	Lansoprazole	Cocrystallization method	21.4 ± 2.3	[80]
γ-CD-MOF	K^+^	γ-CD	Ferulic acid	Cocrystallization method	12.7	[93]
PAA-CD-MOF	K^+^(PAA)	γ-CD	Ibuprofen	Cocrystallization method/impregnation method	12.7/13	[84]
PAA-CD-MOFγ-CD-MOF	K^+^	γ-CD	Lansoprazole	Cocrystallization method/impregnation method	4.5/1.6	[84]
	K^+^	γ-CD	Ibuprofen	Cocrystallization method/impregnation method	23/26	[88]
γ-CD-MOF/C_60_	K^+^ (C_60_)	γ-CD	Doxorubicin	Impregnation method	6.5	[83]
γ-CD-MOF	K^+^	γ-CD	Curcumin	Impregnation method	~	[91]
γ-CD–MOF	K^+^	γ-CD	Captopril/Flurbiprofen	Impregnation method	12.6/12.1	[67]
	~	γ-CD	Salicylic acid/Piroxicam	Impregnation method	9.8/8.44	[67]
	~	γ-CD	Fenbufen/Ketoprofen	Impregnation method	7.97/7.4	[67]
γ-CD-MOF	K^+^ (SNP seeds)	γ-CD	Nile red	Impregnation method	~	[71]
γ-CD-MOF	K^+^	γ-CD	Sucralose	Impregnation method	27.9(Nano)/17.5(Micro)	[90]
γ-CD-MOF	K^+^	γ-CD	Sodium diclofenac	Impregnation method	50	[58]
	Na^+^	γ-CD	Sodium diclofenac	Impregnation method	49	[58]
	Fe^3^	γ-CD	Sodium diclofenac	Impregnation method	55	[58]
CHS-γ-CD-MOF	K^+^	γ-CD	Doxorubicin	Impregnation method	6~8	[97]
γ-CD-MOF	K^+^	γ-CD	Doxorubicin	Impregnation method	6~8	[103]
γ-CD-MOF	K^+^	γ-CD	Fenbufen	Impregnation method	19.6	[78]
γ-CD-MOF	K^+^	γ-CD	Caffeine/theophylline	Impregnation method	~	[104]
VAP-γ-CD-MOF	K^+^	γ-CD	Vitamin A palmitate	Impregnation method	9.77	[105]
β-CD-MOF	Cs^+^ (H3tzdc)	β-CD	5-FU/ Methotrexate	Impregnation method	137.9/68.9	[63]
	Cs^+^ (TsOH)	β-CD	5-FU/ Methotrexate	Impregnation method	151/121.7	[63]
	~	β-CD	5-FU/ Methotrexate	Impregnation method	44.8/79.1	[63]
β-CD-MOF	K^+^	β-CD	Azithromycin	Grinding method	33.91 ± 1.2	[49]
β-CD-MOFβ-CD	Na^+^	β-CD	5-FU	Grinding method	23.02	[59]
	~	β-CD	5-FU	Grinding method	15.73	[59]
β-CD-MOF	Na^+^	β-CD	5-FU/ Quercetin	Grinding method	~	[79]
	K^+^	β-CD	5-FU/ Quercetin	Grinding method	~	[79]
α-CD-MOF	Na^+^	α-CD	5-FU/ Methotrexate	Impregnation method	8.5/38.5	[69]
	Na^+^	α-CD	SL/FA	Impregnation method	12.8/10.87	[69]
	Na^+^	α-CD	QT	Impregnation method	32	[69]
α-CD	~	α-CD	5FU/QT	Impregnation method	6.6/6.83	[69]
	~	α-CD	SL/FA	Impregnation method	3.19/3.0	[69]
	~	α-CD	QT	Impregnation method	2.08	[69]
α-CD-MOF	K^+^	α-CD	5-FU	Impregnation method	25.7	[64]
α-CD-1	~	α-CD	5-FU	Impregnation method	10.7	[64]
α-CD-2	~	α-CD	5-FU	Impregnation method	10.9	[64]

**Table 3:** Pharmacokinetic data for ibuprofen formulations following oral administration to mice (data reprinted with permission from [88]).

Formulation	*C*_max_ (µg/mL)	*T*_max_ (h)	AUC_0–4h_ (µg·h/mL)	AUC_0–00h_ (µg·h/mL)	*T*_1/2_ (h)
IBU K salt	108 ± 4.6	0.28 ± 0.19	144.0 ± 28.2	158.8 ± 37.1	1.07 ± 029
IBU K salt (23 wt %)/γ-CD	224.2 ± 45.7	0.17	273.6 ± 71.1	335.7 ± 122.5	1.46 ± 0.48
CD-MOF/IBU (26 wt %)	93.6 ± 12.5	0.28 ± 0.19	193.4 ± 27.5	290.7 ± 76.3	2.35 ± 0.68
